# Knockdown of Adra2a Increases Secretion of Growth Factors and Wound Healing Ability in Diabetic Adipose-Derived Stem Cells

**DOI:** 10.1155/2022/5704628

**Published:** 2022-11-14

**Authors:** Xiangyuan Zhao, Yong Zhang, Xinzhen Zuo, Shubai Wang, Xiao Dong

**Affiliations:** ^1^College of Life Sciences, Qingdao Agricultural University, Qingdao, Shandong, 266109, China; ^2^College of Animal Science and Technology, Qingdao Agricultural University, Qingdao, Shandong, 266109, China

## Abstract

Studies showed that compared to normal adipose-derived stem cells (ASCs), ASCs from type 2 diabetic (T2D) mice were less effective in treating diabetic cutaneous wounds. However, the mechanisms remain unclear. Our transcriptomic profiling comparison showed that the expression of *α*2A-adrenergic receptor (Adra2a) was significantly increased in ASCs from T2D mice (T2D ASCs). Therefore, the purpose of this study was to investigate whether the elevated Adra2a is involved in the diminished wound-healing capabilities of T2D ASCs. RNA-seq was used to compare the transcriptomic profiles of T2D and normal ASCs. The differential genes were verified by real-time RT-qPCR. Clonidine was used to active Adra2a, and lentivirus-mediated RNAi was used to knockdown Adra2a. The secretion and expression of growth factors were detected by enzyme-linked immunosorbent assay (ELISA) and real-time RT-qPCR, respectively. The cAMP and PKA activity were also detected. Wound healing abilities of various ASCs were assessed in T2D mouse excisional wound models. The results showed Adra2a agonist clonidine decreased the expression and secretion of growth factors, cAMP content, and activity of PKA in ASCs, while Adra2a knockdown T2D ASCs showed the opposite effects. Adra2a knockdown T2D ASCs also showed increased wound-healing capabilities compared to untreated T2D ASCs. Altogether, T2D increased Adra2a expression, which may subsequently decrease the expression and secretion of growth factors and eventually diminish the wound-healing capabilities of T2D ASCs. Adra2a knockdown can restore the secretion of growth factors in T2D ASCs and then accelerate the wound healing, which may provide a new possibility in the treatment of diabetic wounds.

## 1. Introduction

In recent years, adipose-derived stem cells (ASCs), which are obtained from abundant adipose tissue, have been gaining the interest of scientists in the field of cell transplantation [1–3]. The advantages of ASCs include convenient isolation, low immunogenicity, rapid proliferation, and low tumorigenicity [4]. A large number of preclinical and clinical studies have found that ASCs have great potential to treat chronic skin injury, diabetes and diabetic complications, etc. [5–7].

ASCs can secrete cytokines and growth factors such as vascular endothelial growth factor (VEGF), hepatocyte growth factor (HGF), and transforming growth factor-*β* (TGF-*β*) which can promote angiogenesis and antiapoptosis through autocrine or paracrine pathways [8–12]. Our previous studies have found that ASCs can significantly improve chronic pancreatitis [13], survival rate of islets, hyperglycemia, insulin resistance, and wound-healing ability in obese and diabetic mice [10, 14, 15]. We found that the therapeutic effect of ASCs from normal mice is better than ASCs from T2D mice [10, 14].

In order to explore the mechanisms of the lessened therapeutic effect of T2D ASCs, we compared the transcriptomic profiles of T2D and normal ASCs and found that the expression of *α*2A-adrenergic receptor (Adra2a) was significantly increased in T2D ASCs.

Adrenergic receptors belong to a large family of G-protein coupled receptors mediated by catecholamines. According to their different responses to norepinephrine (NE), they are divided into two types: adrenergic *α* receptors and *β* receptors. These two types of receptors are divided into 3 *α*1 (*α*1a, *α*1b, and *α*1d), 3 *α*2 (*α*2a, *α*2b, and *α*2c), and 3 *β* subtypes (*β*1, *β*2, and *β*3) [16, 17]. Studies have shown that overexpression of Adra2a inhibits insulin secretion of pancreatic *β* cells, affects energy and fat metabolism, and plays an important regulatory role in the occurrence of T2D [17, 18]. It is considered as the chief culprit of metabolic syndrome and diabetes [19], but there are few researches on what effects Adra2a has on ASCs.

In this study, we compared the transcriptomic profiles of T2D and normal ASCs and investigated the possible role of Adra2a in the diminished wound-healing capabilities of T2D ASCs.

## 2. Materials and Methods

### 2.1. Animals

Male C57BL/6 mice at 4 weeks of age were purchased from Beijing Vital River Laboratory Animal Technology Co., Ltd. (Beijing, China). The animals were fed and housed in a temperature- and humidity-controlled environment with free access to water and diet under a 12 h light/dark cycle. All animal experiments were approved by the Institutional Animal Care and Use Committee at Qingdao Agricultural University.

### 2.2. Mice Model of Type 2 Diabetes (T2D)

Male C57BL/6 mice were fed for one week to adapt to the new environment. Then, they were randomly divided into two groups and fed with high-fat diet (60% of calories from fat) or standard chow-fat diet (10% of calories from fat) for a total of 20 weeks, respectively. To obtain a mouse model of T2D, after 20 weeks of high-fat diet (HFD) feeding, each mouse was injected with STZ (Sigma-Aldrich, St. Louis, MO, USA) at a daily dose of 40 mg/kg for three consecutive days. The individual body weights and blood glucose levels were measured weekly.

### 2.3. Intraperitoneal Glucose Tolerance Test and Insulin Tolerance Test

Intraperitoneal glucose tolerance test (IPGTT, 2 g/kg of glucose) and insulin tolerance test (ITT, 0.5 unit/kg of insulin) were performed after 21 weeks of high-fat diet as previously described. Blood glucose areas under the curve during an IPGTT and ITT were calculated using the Trapezoidal method [10].

### 2.4. Isolation and Culture of ASCs

Epididymal fat pads were obtained from C57BL/6 or T2D mice and washed with phosphate-buffered saline (PBS). Tissues were digested with 0.1% collagenase type І (Sigma-Aldrich, St. Louis, MO, USA) by incubation in a shaker at 37°C for 15 to 20 minutes. The digestion was terminated by a culture medium containing 10% fetal bovine serum (FBS, Gibco; Langley, OK, USA). The mixture was then centrifuged for 5 min at 1,200 rpm. After centrifugation, the cell pellet was resuspended in complete medium made of Dulbecco's modified Eagle's medium/F12 medium supplemented with 10% FBS and 1% penicillin and streptomycin (Sangon Biotech Co., Ltd., Shanghai, China) [10]. Cells were cultured in 37°C at 5% CO_2_ atmosphere and were passaged at 90% confluence with 0.25% Trypsin-EDTA. The third passage ASCs were used in subsequent experiments.

### 2.5. RNA-Seq

Total RNA was isolated from the third passage of C57BL/6 and T2D ASCs with TRIzol (Invitrogen, Waltham, MA, USA) according to the manufacturer's protocol. The samples were transferred to Beijing Allwegene Technology Co., Ltd. (Beijing, China); Illumina high-throughput sequencing platform (HiSeq 2500/4000) was used for RNA-Seq. After the sequencing images were generated by the sequencing platform, the pixel-level raw data collection, image analysis, and base calling were performed using Illumina Real-Time Analysis software.

### 2.6. RNA Extraction and Real-Time RT-PCR

Total RNA of ASCs was extracted using an RNeasy Kit (Qiagen, Venlo, Netherlands). Reverse transcription was performed using a cDNA Reverse Transcription Kit (TaKaRa Bio, Otsu, Japan). All primers for gene analysis were obtained from Sangon Biotech Co., Ltd. (Shanghai, China). Quantitative expression of *β*-actin in each sample was used as an endogenous control, and relative gene expression was calculated as described previously [10]. Primer sequences are listed in Appendix Table 1.

### 2.7. Stimulation of ASCs with Clonidine

A total of 1 mg clonidine (Sigma-Aldrich, St. Louis, MO, USA) was dissolved in 3.75 ml ddH_2_O to prepare a storage solution. 20 *μ*l and 40 *μ*l storage solutions were added to each 2 ml fresh culture medium containing 0.5% FBS to prepare with clonidine concentrations of 10 *μ*M and 20 *μ*M. Then, the cells were incubated in the prepared medium 6 h, 24 h, 48 h, and 72 h, respectively.

### 2.8. Double Antibody Sandwich Enzyme-Linked Immunosorbent Assay (ELISA)

Clonidine-treated ASCs or untreated ASCs were seeded in serum-free medium in 6-well plates (2 × 10^5^ cells per well) for 5 days. The supernatant of the culture medium was then collected. The concentrations of transforming growth factor-*β* (TGF-*β*), hepatocyte growth factor (HGF), and vascular endothelial growth factor (VEGF) in the supernatant of cells were detected using ELISA kits (Langton Biotechnology Co., Ltd., Shanghai, China) according to the instructions.

### 2.9. cAMP Assay

The cells were incubated with 10 *μ*M clonidine for 5 min, 15 min, and 30 min, at 37°C in 5% CO_2_ environment. To determine the intracellular cAMP levels, cell samples were prepared by 0.1 M HCl, followed by incubation for 20 min at room temperature. The cells were gently scraped off the bottom of the dish and whisked in a pipette. After centrifugation at 10,000 × *g* for 5 min, the supernatants were transferred into new tubes. A cAMP Kinase Activity Assay Kit (BioVision, Inc., Milpitas, CA, USA) was used to detect the content of cAMP in cells.

### 2.10. PKA Assay

The cells were incubated with 10 *μ*M clonidine for 5 min, 15 min, and 30 min at 37°C in 5% CO_2_ environment. 1 ml of lysis buffer was added to a 100 mM culture plate and placed on ice for 10 min. After 13,000 rpm for 5 min, the supernatants were transferred into new tubes and kept on ice. A PKA Kinase Activity Assay Kit (Abcam, Cambridge, MA, USA) was used to detect the activity of PKA in cells. Absorbance was measured using a microplate reader at 450 nM, and the relative kinase activity was calculated according to the instructions.

### 2.11. Lentiviral Transduction

Lentiviral transduction was done with the Lentiviral Transduction Kit (GenePharma, Shanghai, China) according to the manufacturer's protocol. Briefly, ASCs were seeded into 24-well plates in 8 × 10^4^cells/well and then were cultured at 37°C in 5% CO_2_ environment for about 24 h. Lentivirus stock was diluted in fresh medium at 250 MOI, and polybrene was added at a final concentration of 5 *μ*g/ml. When the cells reached about 50-70% confluence, medium in 24-well plates was replaced with lentiviral medium. After 72 h of lentiviral transduction, the cells were observed under a fluorescence inverted microscope. The DNA sequences of the hairpins (21 nucleotides) were shAdra2a1: 5′-TGC TGT TGA CAG TGA GCG AGC AACG TGC TGG TTA TTA TCG TAG TGA AGC CAC AGA TGT ACG ATA ATA ACC AGC ACG TTG CCT GCC TAC TGC CTC GGA-3′; shAdra2a2: 5′-TGC TGT TGA CAG TGA GCG CGC CAC TCA TCT CCA TAG AGA ATA GTG AAG CCA CAG ATG TAT TCT CTA TGG AGA TGA GTG GCG TGC CTA CTG CCT CGG A-3′; and shscramble: 5′-TGC TGT TGA CAG TGA GCG AGC CGC GAT TAG GCT GTT ATA ATA GTG AAG CCA CAG ATG TAT TAT AAC AGC CTA ATC GCG CGG CTT GCC TAC TGC CTC GGA-3′ [20]. The control cells were transducted with the control lentivirus.

### 2.12. Wound-Healing Experiment

T2D mice were anesthetized with pentobarbital and furs were shaved from the upper back. Full-thickness excisional wounds with a diameter of 5 mm were made on both sides of the back spine of the mouse with a puncher. Wound granulation tissues were harvested at 14 days postwounding along with unwounded skin. About 1 cM^2^ of each tissue around the wound and beneath the dermis was collected by careful dissection. The area of the wounds was determined from the digital photos at 1, 4, 7, and 10 days postwounding, and the wound-healing rates were calculated using image software (pixel counts). The rate of wound healing = (wound area on day 1 − wound area on day *n*)/wound area on day 1 × 100%.

### 2.13. Hematoxylin and Eosin (H&E) Staining

H&E staining was performed as described in our previous studies [10, 14]. The granulation tissue and surrounding skin tissues were fixed in 4% paraformaldehyde, gradually dehydrated, embedded in paraffin, and cut into 5 *μ*M sections. Tissue sections were stained with hematoxylin for 10 min, rinsed with water, and then stained with eosin for 1-2 min. Slides were observed using an Olympus BX51 Microscope (Olympus, Tokyo, Japan), and images were captured using an Olympus DP72 Digital Camera. The skin appendages were calculated for statistical analysis.

### 2.14. Statistical Analysis

All experiments were repeated at least three times. Data are presented as mean ± standard derivation (SD). Differences between groups were compared by ANOVA test with Bonferroni correction. ^∗^*P* < 0.05 was considered a significant difference and ^∗∗^*P* < 0.01 was considered an extremely significant difference.

## 3. Results

### 3.1. Modeling of T2D Mice

**Figure 1:**
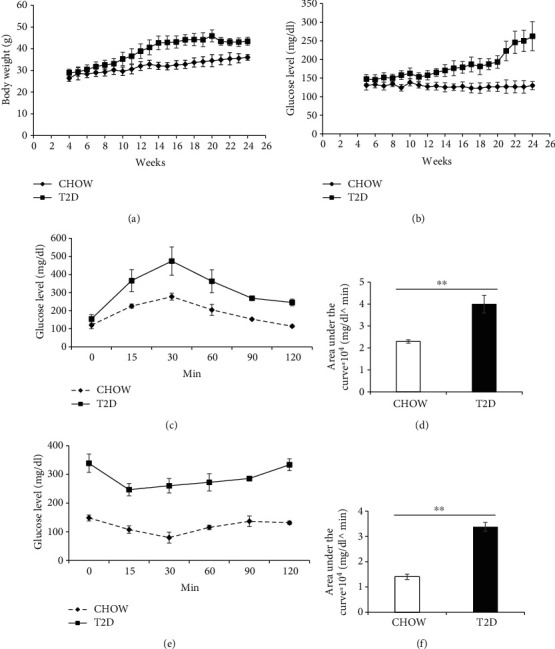
Generation of T2D mice by HFD combined with multiple low-dose STZ injections. (a) Changes in body weights of chow (normal) mice (*n* = 30) and T2D mice (*n* = 40). (b) Blood glucose levels in chow and T2D mice. (c) Blood glucose levels of chow and T2D mice during the IPGTT (*n* = 4 mice per group). (d) Area under the curve during the IPGTT. (e) Blood glucose levels of chow and T2D mice during the ITT (*n* = 4 mice per group). (f) Area under the curve during an ITT. The bars are mean ± SD.^∗^*P* < 0.05; ^∗∗^*P* < 0.01.

To generate T2D mice, 6-week-old C57BL/6 mice were fed with HFD for 20 weeks, the weight of the HFD mice was 45 ± 3.4 g, and the blood glucose increased to 193 ± 8.2 mg/dl, while the weight of the chow mice was 34 ± 1.5 g, and the blood glucose was 126.5 ± 5 mg/dl. Then, HFD mice were injected with STZ; after STZ injection, the weight of the T2D mice decreased significantly and the blood glucose rapidly increased. 4 weeks after STZ injection, the body weight of T2D mice was 43 ± 4 g and their blood glucose was 262 ± 12.5 mg/dl, which remained generally constant (Figures 1(a) and 1(b)). The area under the curve of IPGTT indicates that T2D mice showed impaired glucose tolerance (Figures 1(c) and 1(d)), and the area under the curve of ITT indicates that T2D mice showed increased insulin resistance (Figures 1(e) and 1(f)) compared to chow mice [10]. STZ-treated HFD mice with insulin resistance and blood glucose levels over 200 mg/dl were considered T2D mice.

### 3.2. C57BL/6 and T2D ASC RNA-Seq Results and Global Gene Expression Profiles

**Figure 2:**
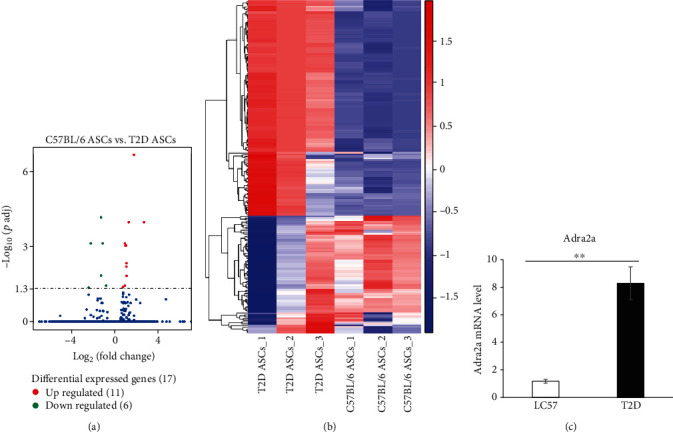
Transcriptomic profiles of C57BL/6 and T2D ASCs. (a) The volcano map shows the overall distribution of the differential genes. Genes with significant differential expression are indicated by red dots (upregulation) and green dots (downregulation). Genes with insignificant differential expression are represented by blue dots (*P* < 0.05). (b) Heat map of gene expression in ASCs from chow mice (*n* = 3) and T2D mice (*n* = 3). Clustering shows twofold up- and down-regulated genes of ASCs. Red and blue represent upregulated and downregulated gene expression, respectively. (c) Expression of Adra2a gene of C57BL/6 and T2D ASCs tested by RT-PCR. ^∗^*P* < 0.05; ^∗∗^*P* < 0.01.

**Figure 3:**
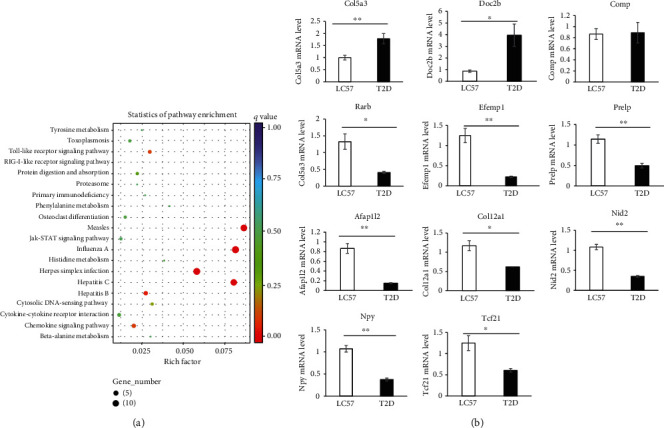
(a) Differential gene KEGG enrichment scatter plot. The size of the dot shows the number of differentially expressed genes in this pathway. (b) Expression of 11 significant differential genes of C57BL/6 and T2D ASCs tested by RT-PCR. ^∗^*P* < 0.05; ^∗∗^*P* < 0.01.

After they were passaged 3 times, C57BL/6 and T2D ASCs were long, spindle-like, densely arranged, and interwoven in bunches with no obvious difference in shape (Supplementary Figure 1A). T2D ASCs proliferated slower than C57BL/6 ASCs (Supplementary Figure 1B). These two groups of ASCs have the potential to differentiate into adipocytes, bone cells, and chondrocytes, and the specific details of flow cytometry were referred to our previous article [10]. We made a transcriptome sequencing analysis of the third passage of ASCs from C57BL/6 and T2D mice and found 17 differential genes, of which 6 are upregulated and 11 are downregulated in T2D ASCs compared to C57BL/6 ASCs (Figure 2(a)). The heat map analysis of the global genes is as follows in Figure 2(b). The KEGG pathway enrichment analysis of differential genes revealed that these differential genes are mainly enriched in collagen (Col5a3 and Col12a1) digestion and absorption, cAMP signaling pathway, regulation of lipolysis in adipocytes, adrenaline receptor- (Adra2a-) related neuronal receptor interactions and ECM receptor-related signaling pathways (Figure 3(a)). RT-PCR was used to validate the reliability of the RNA-seq results. There are 11 genes that showed significant difference by RT-PCR. The expression levels of Adra2a (Figure 2(c)), Col5a3, and Doc2b genes of T2D ASCs were significantly higher than C57BL/6 ASCs, and the gene expression levels of Rarb, Efemp1, Prelp, Afap1l2, Col12a1, Nid2, Npy, and Tcf21 were significantly lower in T2D ASCs than C57BL/6 ASCs (Figure 3(b)). These results are identical to RNA-seq.

### 3.3. Effects of Adra2a on the Secretion of Growth Factors in ASCs

**Figure 4:**
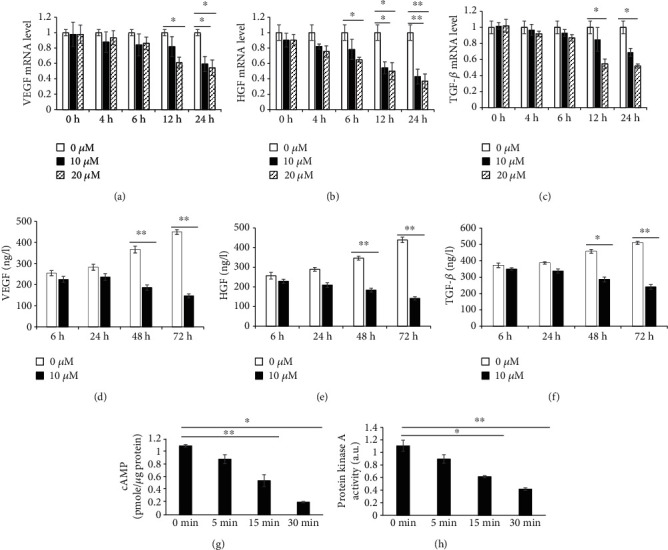
Clonidine reduces the expression and secretion of growth factors in C57BL/6 ASCs. The mRNA expression levels of VEGF (a), HGF (b), and TGF-*β* (c) at 0 h, 4 h, 6 h, 12 h, and 24 h of C57BL/6 ASCs treated with 10 *μ*M and 20 *μ*M of clonidine, respectively. Secretion of VEGF (d), HGF (e), and TGF-*β* (f) at 6 h, 24 h, 48 h, and 72 h of C57BL/6 ASCs treated with 10 *μ*M clonidine. Clonidine reduces contents of cAMP and PKA in C57BL/6 ASCs. The contents of cAMP (g) and PKA (h) at 0 min, 5 min, 15 min, and 30 min with 20 *μ*M clonidine treatment. Three biological replicates and three technical replicates were used in each case. ^∗^*P* < 0.05; ^∗∗^*P* < 0.01.

After treatment with Adra2a agonist (clonidine) at 10 *μ*M and 20 *μ*M for 0 h, 4 h, 6 h, 12 h, and 24 h, the expression levels of VEGF, HGF, and TGF-*β* in ASCs were detected (Figures 4(a)–4(c)). The results showed that after 12 h and 24 h of clonidine treatment, the expression levels of all three growth factors in ASCs decreased. The difference in HGF expression appeared earlier. After 6 hours, the expression level of HGF in 20 *μ*M clonidine-treated ASCs was lower than in untreated ASCs (*P* < 0.05). The difference between TGF-*β* and VEGF was more obvious after 12 hours (*P* < 0.05).

The secretion levels of the three growth factors are shown in Figures 4(d)–4(f). The stimulatory effect of clonidine was evident also after 48 h and 72 h treatment. The secretion of VEGF and HGF decreased extremely significantly (*P* < 0.01), while TGF-*β* decreased significantly (*P* < 0.05) in clonidine-treated ASC supernatant compared to clonidine-untreated ASC supernatant after 48 h treatment. The three growth factors all decreased extremely significantly (*P* < 0.01) in clonidine-treated ASC supernatants compared to clonidine-untreated ASC supernatant after 72 h treatment. There was no significant difference in the secretion of growth factors before 24 h treatment.

In addition to this data, the pathway by which Adra2a inhibits growth factor secretion by modulating cAMP levels in ASCs is detected. We collected ASCs treated with clonidine for 0 min, 5 min, 15 min, and 30 min, respectively. The results indicated that the amount of cAMP (Figure 4(g)) and PKA activity (Figure 4(h)) reduced significantly after 15 min (*P* < 0.05) and 30 min (*P* < 0.01) of clonidine treatment, respectively.

### 3.4. Effects of Adra2a Knocking Down on ASCs

**Figure 5:**
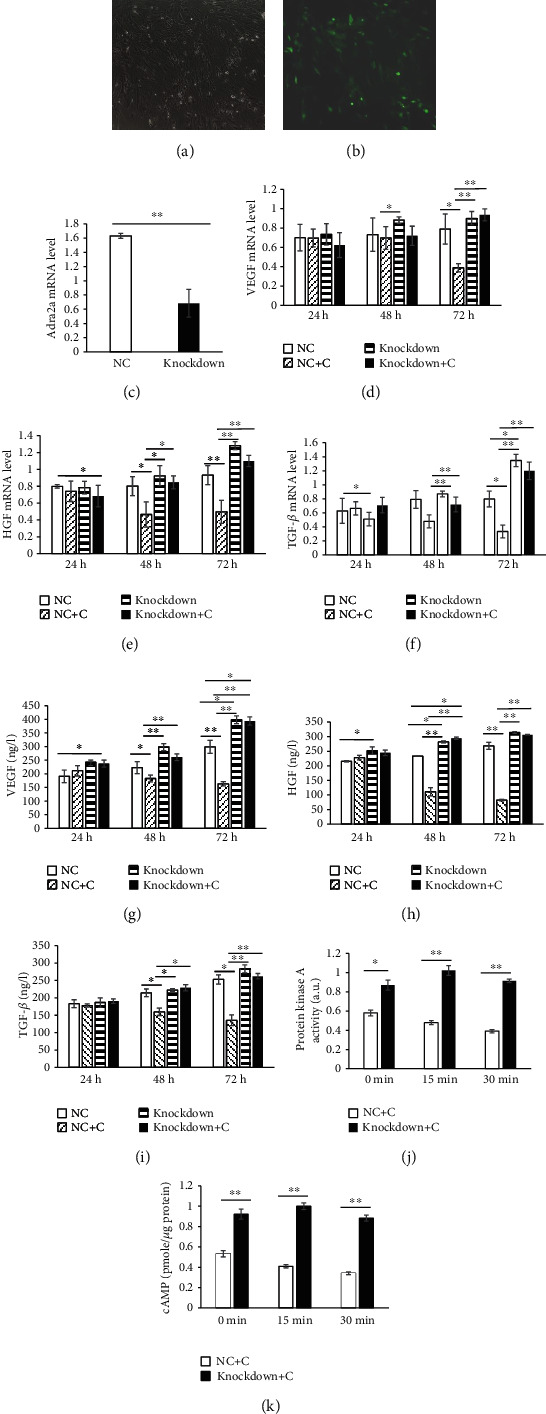
Adra2a knocking down increases the expression and secretion of growth factors in T2D ASCs. Representative micrographs of T2D ASCs and T2D ASCs with lentivirus. Green represents lentivirus successfully transfected into the cells (a, b). The levels of expression of Adra2a from T2D ASCs transfected with Adra2a knockdown lentivirus and negative control lentivirus (c). The expression of VEGF (d), HGF (e), and TGF-*β* (f) in the NC, NC+C, knockdown, and knockdown+C group at 24 h, 48 h, and 72 h, respectively. The secretion of VEGF (g), HGF (h), and TGF-*β* (i) in the NC, NC+C, knockdown, and knockdown+C group at 24 h, 48 h, and 72 h, respectively. The change of cAMP and PKA in T2D ASCs after Adra2a knocking down (j, k): the contents of cAMP (j) and activities of PKA (k) at 0 min, 5 min, and 15 min with 10 *μ*M clonidine treatment. Note: NC: Negative control, T2D ASCs transfected with control vectors; knockdown: Adra2a knocked-down T2D ASCs; NC+C: Negative control T2D ASCs treated with 10 *μ*M clonidine; knockdown+C: Adra2a knocked-down T2D ASCs treated with 10 *μ*M clonidine. Three biological replicates and three technical replicates were used in each case. ^∗^*P* < 0.05; ^∗∗^*P* < 0.01.

To verify whether Adra2a caused a series of changes in growth factors in ASCs, we knocked down T2D ASC Adra2a by lentiviral transduction to prevent clonidine mediated suppression of ASCs. The lentivirus is packaged with green fluorescent protein (GFP), which can be transferred into T2D ASCs along with the lentivirus. The expression of the Adra2a gene in the lentivirus infection T2D ASCs was significantly lower than that of the negative control group (*P* < 0.01) (Figures 5(a)–5(c)).

The third passage T2D ASCs were randomized into T2D ASCs, namely, the negative control (NC) group, Adra2a knockdown T2D ASC (knockdown) group, T2D ASCs treated with 10 *μ*M clonidine (NC+C) group, and Adra2a knockdown T2D ASCs treated with 10 *μ*M clonidine (knockdown+C) group. Three-time periods of 24 h, 48 h, and 72 h were set to detect the differences in the expression of VEGF, HGF, and TGF-*β* in the cells. The results showed that, at 24 h, the expression levels of the three growth factors in the 4 groups were generally the same, but at 48 h and 72 h, the expression levels of TGF-*β* in the NC, knockdown+C, and knockdown group were higher than those of the NC+C group, and the difference was extremely significant (*P* < 0.01), while the expression of HGF and VEGF had an extremely significant difference after 72 h (*P* < 0.01). This indicated that after Adra2a was knocked down, the effect of clonidine on ASCs was eliminated (Figures 5(d)–5(f)).

At the same time, we set three time periods of 24 h, 48 h, and 72 h to detect the difference in the secretion of VEGF, HGF, and TGF-*β* in T2D ASCs. The results showed that at 24 h, the secretion of the three growth factors was relatively the same in the 4 groups, but at 48 h and 72 h, the secretion of the three growth factors increased significantly in the NC, knockdown, and knockdown+C groups. This indicates that after Adra2a is knocked down, clonidine has little effect on the Adra2a of T2D ASCs and the secretion of the three growth factors of the NC, knockdown, and knockdown+C groups were significantly higher than that of the NC+C group (Figures 5(g)–5(i)).

In addition, the T2D ASC (NC+C) and Adra2a knocked-down T2D ASC (knockdown+C) groups were treated with 10 *μ*M clonidine for 0 min, 15 min, and 30 min, respectively. We found that after the Adra2a gene expression was knocked down, the cAMP content and PKA activity in the knockdown+C group was significantly higher than those in the NC+C group under the stimulation of clonidine (*P* < 0.01) (Figures 5(j) and 5(k)).

### 3.5. Effects of Adra2a Knocked-Down T2D ASCs on Wound Healing in T2D Mice

**Figure 6:**
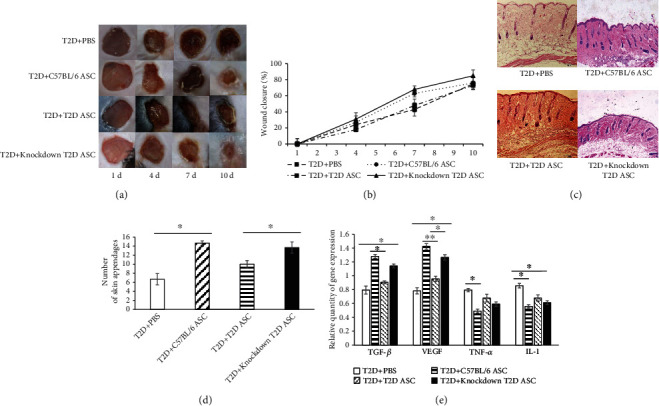
Effects of Adra2a knocked-down T2D ASCs on wound healing in T2D mice. (a) Macroscopic pictures of representative wounds of T2D mice treated with PBS, C57BL/6 ASCs, T2D ASCs, and Adra2a knocked-down T2D ASCs (namely, T2D+PBS; T2D+C57BL/6 ASC; T2D+T2D ASC; T2D+knocked-down T2D ASC groups, respectively, same as below) on day 1, 4, 7, and 10 after wounding. (b) Graphical summary of changes in wound area expressed relative to the initial size of the wound at day 1. Data are mean ± SD. (c) H&E staining of wound sections of T2D mice treated with PBS, C57BL/6 ASCs, T2D ASCs, and Adra2a knocked-down T2D ASCs 14 days postwounding. (d) Statistics of the number of hair follicles around the wound in the skin of T2D mice. (e) The expression of VEGF, TGF-*β*, TNF-*α*, and IL-1in the tissue of the wound area treated with PBS, C57BL/6 ASCs, T2D ASCs, and Adra2a knocked-down T2D ASCs on the 14th day.

T2D mice were randomly transplanted with PBS (T2D+PBS), C57BL/6 ASCs (T2D+C57BL/6 ASCs), T2D ASCs (T2D+T2D ASCs), and Adra2a knocked-down T2D ASCs (T2D+knockdown T2D ASCs) (*n* = 3) after wounding. The wound area was analyzed as a measure of wound healing. The initial expansion of the wound after surgery was very limited in the Adra2a knockdown T2D ASC-treated mice versus the PBS-treated. On day 10, Adra2a knocked-down T2D ASC-treated T2D mice showed a significant enhancement in wound healing (Figure 6(a)). The healing rates of C57BL/6 ASCs and Adra2a knocked-down T2D ASC-treated mice were higher than the PBS and T2D ASC treatment groups (Figure 6(b)). Granulation tissue around the wound was extracted from T2D mice in the four groups and made into paraffin sections for hematoxylin and eosin staining. Compared to the T2D+PBS group, the numbers of hair follicles around the wound were higher in the remaining groups. Among them, the T2D+C57BL/6 ASC and T2D+knockdown T2D ASC groups was significantly exceeding the T2D+PBS and T2D+T2D ASC groups (*P* < 0.05), but the difference between the T2D+C57BL/6 ASC and T2D+Knockdown T2D ASC groups was not significant (Figures 6(c) and 6(d)). The tissue of the wound area was collected at the end of the study. By detecting the expression of growth factors and inflammatory factors in the 4 groups of T2D mice on the 14th day, we found that the relative expression of VEGF and TGF-*β* in the T2D+C57BL/6 ASC and T2D+knockdown T2D ASC groups was higher than the PBS and T2D ASC treatment groups. However, the expression of TNF-*α* and IL-1 in the T2D+C57BL/6 ASC and T2D+knockdown T2D ASC groups was lower than the PBS and T2D ASC treatment groups, respectively (Figure 6(e)).

## 4. Discussion

Our previous research had shown that intravenous infusion of C57BL/6, T2D, or db/db ASCs reduced blood glucose levels and improved insulin sensitivity in T2D mice [10]. In addition, we also found that ASCs from diabetic and normal mice could promote wound healing in diabetic mice [14, 21]. However, there are some differences in the treatment of transplantation between ASCs derived from diabetic and normal mice [10, 14, 21]. Presently, we found some differential genes related to cell proliferation, differentiation, and pathogenesis through RNA-seq of ASCs in normal and T2D mice, including Adra2a, Rarb, Nid2, Afap1l2, Npy, and Tcf21. These differential genes are mainly concentrated in collagen digestion and absorption, cAMP signaling pathways, neuropeptide-related regulation of lipolysis in adipocytes, neuronal receptor interactions related to adrenergic receptors, and ECM receptor-related signaling pathways, etc.

Adra2a mediates adrenergic suppression of insulin secretion. Researches have shown that overexpression of Adra2a in *β* cells can lead to impaired insulin secretion and glucose intolerance and increase the risk of T2D [18, 22, 23]. Pharmacological receptor antagonism, silencing of receptor expression, or blockade of downstream effectors rescued insulin secretion in congenic islets [23, 24]. People found that the fasting glucose of rs553668A carriers was significantly higher than that of noncarriers, and they have reduced insulin secretion and higher Adra2a mRNA expression, and this increased the risk of T2D [18, 25, 26]. However, the effect of Adra2a on ASCs was still lacking in understanding both in mice and humans.

ASCs can promote tissue regeneration by secreting cytokines and growth factors [27–30]. Studies have confirmed that HGF plays an important role in acute injury and regeneration [10, 21, 30]. VEGF can not only promote the angiogenesis of damaged tissues but also inhibit the accumulation of ectopic lipids, restore peripheral insulin sensitivity, and maintain islet function [31]. TGF-*β*, which belongs to the TGF cytokine superfamily, can regulate cell migration, cell adhesion, and extracellular matrix synthesis and promote the proliferation, differentiation, and apoptosis of a variety of cells. It plays an important role in bone reconstruction, wound repair, and embryonic development [32, 33]. We have found that in terms of VEGF, HGF, and TGF-*β* secretion ability, ASCs derived from T2D mice are inferior to ASCs derived from normal mice especially in the ability to secrete HGF [10]. In this study, we used Adra2a agonist clonidine to treat ASCs at different time and found that the expression and secretion of these three growth factors decreased significantly and the cAMP content and PKA activity downstream of the pathway was also affected. We used lentivirus-mediated RNA interference to decrease the expression of Adra2a in T2D ASCs to determine the specific function of this receptor in ASCs and found that the expression and secretion of growth factors in Adra2a knocked-down T2D ASCs were significantly higher than that of T2D ASCs, and after Adra2a knockdown, the T2D ASC treatment groups did not work on the stimulation of clonidine. In the meantime, the content of cAMP and the activity of PKA had also increased. Therefore, it may be that the microenvironment such as higher glycemia and abnormal insulin and epinephrine in T2D mice affects the growth factor secretion pathway of ASCs [17–19, 34–37], which in turn affects the expression and secretion capacity of growth factors in ASCs, resulting in differences in transcriptional regulation.

Finally, we designed in vivo experiments to investigate the effect of T2D ASCs on wound healing after Adra2a knocked down. We found that the wound-healing rate, the growth of hair follicles around the wound, and the expression of growth factors were increased while inflammatory factors decreased after Adra2a knocked-down T2D ASC transplantation in the in vivo experiments, and the results further demonstrated that Adra2a knocked-down T2D ASCs showed increased cutaneous wound-healing capabilities compared to untreated T2D ASCs.

In addition, from our results, the cAMP levels and PKA activities of ASCs decreased significantly after treated with clonidine for 15 min, the mRNA expression of the three growth factors decreased significantly after 12 h, while the secretion of the three growth factors decreased significantly after 48 h (Figure 4). There is an opposite trend after Adra2a were knocked down in T2D ASCs (Figure 5). Therefore, it is maybe that the decreased activation of cAMP/PKA caused a decrease in growth factor expression and then resulted in a decrease in growth factor secretion in ASCs.

**Figure 7:**
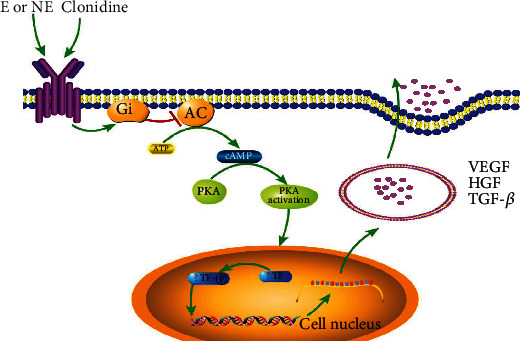
Regulation of growth factor exocytosis by Adra2a. Activation of these receptors by epinephrine (E), norepinephrine (NE), and clonidine in ASCs results in receptor-Gi coupling that inhibits adenyl cyclase (AC). This inhibition, in turn, results in decreased conversion of ATP to cAMP and reduced protein kinase A (PKA) activity; this may increase the activation of some transcription factor- (TF-) mediated mRNA expression of growth factors and then decline the exocytosis of the growth factors in ASCs.

From these results, we speculate that activation of Adra2a by epinephrine (E), norepinephrine (NE), clonidine, etc. in ASCs results in receptor-Gi coupling that inhibits adenyl cyclase (AC). This inhibition, in turn, results in the decreased conversion of ATP to cAMP and reduced protein kinase A (PKA) activity; this may affect the activation of some transcription factors which mediate the mRNA expression of growth factors and then decline the exocytosis of the growth factors, which is an important reason for the decreased secretion of growth factors in T2D ASCs (Figure 7).

This study discovered that Adra2a can affect growth factor secretion of ASCs for the first time, although the mechanisms remain unclear. We investigated some downstream molecules of the signaling pathway which may contribute to the decrease of growth factor secretion in T2D ASCs. The mechanisms of the upregulation of Adra2a in T2D ASCs are still unclear; the effect of Adra2a on ASCs was still lacking of understanding in humans and more research is needed,

## 5. Conclusion

In summary, these findings demonstrate that the overexpression of Adra2a can reduce the secretion of growth factors in ASCs, and knocking down Adra2a in T2D ASCs can restore the ability of T2D ASCs to secrete growth factors, which provides a possibility to shrink the difference of normal and T2D ASCs in clinical studies. This study will provide possibilities and create a new theoretical basis for the treatment of T2D and complications by ASC autologous transplantation in patients with T2D.

## Data Availability

The data used and/or analyzed during the current study are available from the corresponding authors on reasonable request.

## Supplementary Materials

Supplementary Materials Supplementary Figure 1: (A) the representative micrographs of C57BL/6 and T2D ASCs at passage 3 observed under a light microscope. (B) Growth curve of C57BL/6 and T2D ASCs at passage 3. Supplementary Tables. Appendix Table 1: primer sequences.
