# Supplementary material for: Knockdown of Adra2a Increases Secretion of Growth Factors and Wound Healing Ability in Diabetic Adipose-Derived Stem Cells
Source: Stem Cells Int. 2022 Nov 14;2022:5704628. doi: 10.1155/2022/5704628 (PMC9678456; doi:10.1155/2022/5704628)
Supplement: Supplementary Materials — Supplementary Figure 1: (A) the representative micrographs of C57BL/6 and T2D ASCs at passage 3 observed under a light microscope. (B) Growth curve of C57BL/6 and T2D ASCs at passage 3. Supplementary Tables. Appendix Table 1: primer sequences. [file 5704628.f1.zip › 1 20221019 Zhao supplementary table.docx]

**Appendix Table 1. Primer sequences**

| **Gene** | | **Primers** | **Size** | **Accession** |
| --- | --- | --- | --- | --- |
| *β-actin*  *Col5a3*  *Doc2b*  *Comp*  *Adra2a*  *Rarb*  *Efemp1*  *Afap1l2*  *Npy*  *Prelp*  *Nid2* | F:GGCTGTATTCCCCTCCATCG  R:CCAGTTGGTAACAATGCCATGT  F:CGGGGTACTCCTGGTCCTAC  R:GCATCCCTACTTCCCCCTTG  F:CGACGGCTACGAGTCAGAC  R:TTCAGGGTGTTCCGAAGAGTT  F:ACTGCCTGCGTTCTAGTGC  R:CGCCGCATTAGTCTCCTGAA  F:GTGACACTGACGCTGGTTTG  R:CCAGTAACCCATAACCTCGTTG  F:GCAGTGCGTGGACACATGA  R:GGCAGGGAGAGTCCTCTGAT  F:GCGCTGGTCAAGTCACAGTA  R:AAGCATCTGGGACAATGTCAC  F: CTCCTGCGGCTTTACACCAA  R: TTTGTTCCCCGTTGACTGACA  F: GGGGCCGGACTGTATTTACT  R:ATGCTAGGTAACAAGCGAATGG  F:TGGCTCCTCCCACTTCTCC  R:CGTGGACAGTCAGGGAAGAC  F:TGGATTACCCAATGGATTGACCT  R:GTGGTTTTGGATGACACGTCG | | 154 bp  118bp  225bp  120bp  204 bp  83 bp  127 bp  82 bp  161 bp  194 bp  124 bp | NM_007393.5  NM_001317388.1  NM_007873.3  NM_016685.2  NM_007417.4  NM_001289760.1  NC_000077.6  NM_001177797.1  NM_023456.3  NM_054077.4  NC_000080.6 |
| *Col12a1*  *Oxtr*  *Kprp* | F:AAGTTGACCCACCTTCCGAC  R:GGTCCACTGTTATTCTGTAACCC  F:GATCACGCTCGCCGTCTAC  R:CCGTCTTGAGTCGCAGATTC  F:AACCCGTTCGTTGTCCCAG  R:TTGGGTGAAGTTATATGAGCCAC | | 111 bp  98 bp  134 bp | NM_001290308.1  NM_001081147.2  NM_001002290.1 |
| *VEGF*  *HGF*  *TGF-β* | F:5'-CTGCCGTCCGATTGAGACC-3’  R:5'-CCCCTCCTTGTACCACTGTC-3’  F:5'-ATGTGGGGGACCAAACTTCTG -3’  R:5'-GGATGGCGACATGAAGCAG -3’  F:5'-CTCCCGTGGCTTCTAGTGC -3’  R:5'-GCCTTAGTTTGGACAGGATCTG -3’ | | 233 bp  79bp  114 bp | NM_001025250.3  NM_001289458.1  NM_011577.2 |
