# Supplementary material for: Knockdown of Adra2a Increases Secretion of Growth Factors and Wound Healing Ability in Diabetic Adipose-Derived Stem Cells
Source: Stem Cells Int. 2022 Nov 14;2022:5704628. doi: 10.1155/2022/5704628 (PMC9678456; doi:10.1155/2022/5704628)
Supplement: Supplementary Materials — Supplementary Figure 1: (A) the representative micrographs of C57BL/6 and T2D ASCs at passage 3 observed under a light microscope. (B) Growth curve of C57BL/6 and T2D ASCs at passage 3. Supplementary Tables. Appendix Table 1: primer sequences. [file 5704628.f1.zip › 1 20221019 Zhao suppl figure.docx]

**Supplementary figure1**

**
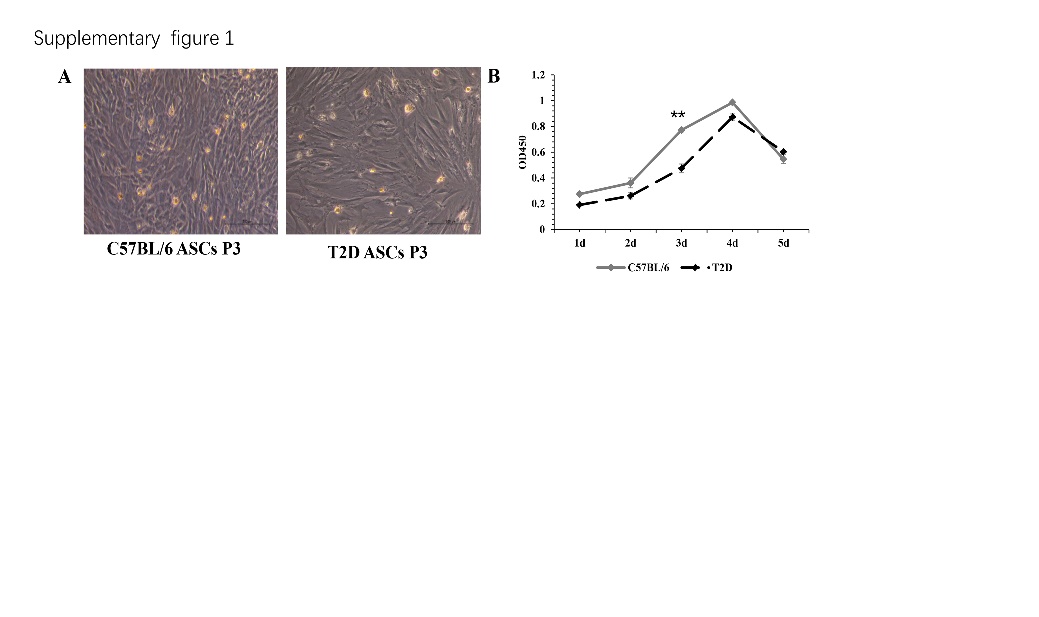
**

**Legend：**

**Supplementary figure1：**(A) The representative micrographs of C57BL/6 and T2D ASCs at passage 3 observed under a light microscope. (B) Growth curve of C57BL/6 and T2D ASCs at passage 3.
